# Prevalence of Obesity Among Adults, by Household Income and Education — United States, 2011–2014

**DOI:** 10.15585/mmwr.mm6650a1

**Published:** 2017-12-22

**Authors:** Cynthia L. Ogden, Tala H. Fakhouri, Margaret D. Carroll, Craig M. Hales, Cheryl D. Fryar, Xianfen Li, David S. Freedman

**Affiliations:** ^1^Division of Health and Nutrition Examination Surveys, National Center for Health Statistics, CDC; ^2^Office of Analysis and Epidemiology, National Center for Health Statistics, CDC; ^3^Divsion of Nutrition, Physical Activity and Obesity, National Center for Chronic Disease Prevention and Health Promotion, CDC.

Studies have suggested that obesity prevalence varies by income and educational level, although patterns might differ between high-income and low-income countries (*1*–*3*). Previous analyses of U.S. data have shown that the prevalence of obesity varied by income and education, but results were not consistent by sex and race/Hispanic origin (*4*). Using data from the National Health and Nutrition Examination Survey (NHANES), CDC analyzed obesity prevalence among adults (aged ≥20 years) by three levels of household income, based on percentage (≤130%, >130% to ≤350%, and >350%) of the federal poverty level (FPL) and individual education level (high school graduate or less, some college, and college graduate). During 2011–2014, the age-adjusted prevalence of obesity among adults was lower in the highest income group (31.2%) than the other groups (40.8% [>130% to ≤350%] and 39.0% [≤130%]). The age-adjusted prevalence of obesity among college graduates was lower (27.8%) than among those with some college (40.6%) and those who were high school graduates or less (40.0%). The patterns were not consistent across all sex and racial/Hispanic origin subgroups. Continued progress is needed to achieve the *Healthy People 2020* targets of reducing age-adjusted obesity prevalence to <30.5% and reducing disparities (*5*).

NHANES is a biannual cross-sectional survey designed to monitor the health and nutritional status of the civilian noninstitutionalized U.S. population (*6*). The survey consists of in-home interviews and standardized physical examinations conducted in mobile examination centers. During the physical examination, standardized measurements of weight and height were obtained. Body mass index (BMI) was calculated as weight in kilograms divided by height in meters squared. Obesity was defined as a BMI ≥30 kg/m^2^. The NHANES sample is selected through a complex, multistage probability design. Participants self-reported race/Hispanic origin, and were divided into five categories: non-Hispanic white, non-Hispanic black, non-Hispanic Asian, Hispanic and “other.” During 2011–2014, non-Hispanic black, non-Hispanic Asian, and Hispanic persons, among other groups, were oversampled. A total of 308 non-Hispanic persons reporting other races or more than one race were placed in an “other” category, and their data were included in the overall results. The NHANES examination response rate for adults aged ≥20 years was 64.5% in the 2011–2012 survey and 63.7% in the 2013–2014 survey.

Household income was categorized using FPL information, which accounts for inflation and family size (https://aspe.hhs.gov/prior-hhs-poverty-guidelines-and-federal-register-references); income levels were designated as ≤130%, >130% to ≤350%, and >350% of FPL. The cut point for participation in the Supplemental Nutrition Assistance Program is 130% of the poverty level, and 350% provides relatively equal sample sizes for each of the three income groups. Education was categorized as high school graduate or less, some college, and college graduate.

All estimates were adjusted to account for the complex survey design, including examination sample weights. Estimates were age-adjusted to the 2000 projected U.S. Census population using the age groups 20–39, 40–59, and ≥60 years. Confidence intervals for estimates were calculated using the Wald method. Differences between income and education groups were tested using a two-sided, univariate t-statistic, with statistical significance defined as a p-value of <0.05. Temporal trends from 1999–2002 to 2011–2014 were analyzed using orthogonal contrasts and 2-year survey cycles. Pregnant women (122) and participants with missing weight or height (571) were excluded, resulting in a total sample size of 10,636 for the period 2011–2014. For estimates by FPL, an additional 851 participants were excluded because of missing FPL data, and for estimates by education, eight participants were excluded because information on education was missing.

During 2011–2014, the age-adjusted prevalence of obesity was 38.3% among women and 34.3% among men (Table). The prevalence of obesity was 34.5% among non-Hispanic white adults, 48.1% among non-Hispanic black adults, 11.7% among non-Hispanic Asian adults, and 42.5% among Hispanic adults.

**TABLE:** Prevalence of obesity among adults,* by race/Hispanic origin, sex, household income (percentage of FPL), and education — National Health and Nutrition Examination Survey, 2011–2014

Characteristic	No.	Race/Hispanic origin				
		Overall	White, non-Hispanic	Black, non-Hispanic	Asian, non-Hispanic	Hispanic
		% (95% CI)	% (95% CI)	% (95% CI)	% (95% CI)	% (95% CI)
Overall	10,636	36.3 (34.7–38.0)	34.5 (32.4–36.7)	48.1 (45.5–50.7)	11.7 (9.8–13.7)	42.5 (39.8–45.3)
Women	5,413	38.3 (36.1–40.5)	35.5 (32.4–38.6)	56.9 (54.2–59.7)	11.9 (8.8–15.1)	45.7 (42.2–49.2)
Men	5,223	34.3 (32.6–36.1)	33.6 (31.4–35.7)	37.5 (34.3–40.8)	11.2 (8.8–13.6)	39.0 (35.4–42.5)
**Household income, both sexes**						
≤130% FPL	3,462	39.0 (36.9–41.0)	35.8 (32.8–38.7)	46.6 (43.2–50.0)	15.0 (9.7–20.3)	42.6 (38.1–47.1)
>130 to ≤350% FPL	3,331	40.8 (38.2–43.4)	40.2 (36.5–43.9)	48.8 (44.6–52.9)	11.2 (6.6–15.8)	45.0 (40.7–49.2)
>350% FPL	2,992	31.2 (28.3–34.2)^†,§^	30.6 (27.3–34.0)^†,§^	49.3 (43.4–55.1)	10.7 (8.3–13.1)	39.1 (33.9–44.3)
**Household income, women**						
≤130% FPL	1,835	45.2 (42.5–48.0)	42.0 (37.4–46.5)	55.8 (52.2–59.4)	17.2 (10.3–24.1)	48.7 (43.1–54.4)
>130 to ≤350% FPL	1,702	42.9 (40.1–45.8)	42.5 (38.8–46.1)	59.4 (53.7–65.2)	11.7 (5.6–17.7)	44.6 (37.4–51.8)
>350% FPL	1,453	29.7 (26.1–33.3)^†,§^	27.9 (24.0–31.9)^†,§^	56.7 (50.0–63.5)	9.7 (5.8–13.7)	42.9 (35.2–50.5)
**Household income, men**						
≤130% FPL	1,627	31.5 (28.5–34.4)	28.5 (24.4–32.6)	33.8 (28.9–38.6)	11.8 (4.7–18.9)	35.9 (30.9–40.8)
>130 to ≤350% FPL	1,629	38.5 (35.1–41.9)^†^	37.8 (32.7–43.0)^†^	35.6 (30.7–40.5)	10.3 (5.6–15.0)	44.6 (40.1–49.2)^†^
>350% FPL	1,539	32.6 (29.4–35.8)^§^	32.9 (29.2–36.6)	42.7 (35.8–49.6)^†^	11.8 (7.9–15.7)	35.6 (27.8–43.4)^§^
**Education, both sexes**						
High school graduate or less	4,714	40.0 (37.9–42.2)	38.1 (34.5–41.6)	46.6 (42.8–50.4)	11.5 (7.6–15.5)	43.8 (40.6–47.0)
Some college	3,231	40.6 (38.1–43.1)	39.2 (35.9–42.5)	50.5 (46.3–54.7)	12.4 (8.9–15.8)	42.9 (38.2–47.5)
College graduate	2,683	27.8 (25.0–30.7)^¶,^**	27.5 (24.1–30.9)^¶,^**	47.3 (43.3–52.1)	11.1 (8.7–13.6)	36.9 (30.6–43.2)^¶^
**Education, women**						
High school graduate or less	2,277	45.3 (42.3–48.3)	43.3 (38.7–47.8)	57.9 (53.2–62.6)	11.4 (6.1–16.7)	49.6 (45.6–53.7)
Some college	1,777	41.2 (38.5–43.9)	38.9 (35.1–42.7)	58.8 (53.8–63.9)	13.3 (7.6–19.0)	43.0 (36.3–49.8)
College graduate	1,355	27.8 (24.1–31.5)^¶,^**	27.0 (22.3–31.6)^¶,^**	52.1 (47.4–56.8)**	11.3 (7.6–15.0)	36.1 (26.5–45.6)^¶^
**Education, men**						
High school graduate or less	2,437	35.5 (33.0–37.9)	34.1 (29.7–38.5)	36.0 (30.7–41.2)	11.0 (5.7–16.2)	37.7 (34.0–41.4)
Some college	1,454	40.0 (35.9–44.1)	39.9 (34.7–45.1)	38.2 (32.7–43.7)	10.3 (5.6–15.1)	42.9 (36.0–49.9)
College graduate	1,328	27.9 (24.3–31.5)^¶,^**	28.1 (24.1–32.1)**	40.4 (32.4–48.3)	11.0 (7.9–14.1)	38.5 (28.1–48.8)

**Abbreviations:** CI = confidence interval; FPL = federal poverty level.

*Age-adjusted by the direct method to the 2000 projected U.S. Census population using the age groups 20–39, 40–59, and ≥60 years.

^†^ Significantly different from ≤130% FPL, p<0.05.

^§^ Significantly different from >130 to ≤350% FPL, p<0.05.

^¶^ Significantly different from high school graduate or less, p<0.05.

** Significantly different from some college, p<0.05.

Among women, prevalence was lower in the highest income group (29.7%) than in the middle (42.9%) and lowest (45.2%) income groups. This pattern was observed among non-Hispanic white, non-Hispanic Asian, and Hispanic women, but it was only significant for white women. Among non-Hispanic black women, there was no difference in obesity prevalence among the income groups.

Among men, the prevalence of obesity was lower in both the lowest (31.5%) and highest (32.6%) income groups compared with the middle-income group (38.5%). This pattern was seen among both non-Hispanic white and Hispanic men, although among non-Hispanic white men, the difference between the highest-income and middle-income groups was not statistically significant. Among non-Hispanic black men, obesity prevalence was higher in the highest income group (42.7%) than in the lowest income group (33.8%). There was no difference in obesity prevalence by income among non-Hispanic Asian men.

In 2011–2014, the prevalence of obesity was lower among women and men who were college graduates (27.8% [women], 27.9% [men]) than among women and men with some college (41.2%, 40.0%) and women and men who were high school graduates or less (45.3%, 35.5%). By race/Hispanic origin, the same pattern was seen among non-Hispanic white, non-Hispanic black, and Hispanic women, and also among non-Hispanic white men, although the differences were not all statistically significant. Although the difference was not statistically significant among non-Hispanic black men, obesity prevalence increased with educational attainment. Among non-Hispanic Asian women and men and Hispanic men there were no differences in obesity prevalence by education level.

From 1999–2002 to 2011–2014 the prevalence of obesity increased among women in the two lower income groups, but not among women living in households with incomes above 350% of FPL. Obesity prevalence increased among men in all three income groups during this period (Figure 1). Obesity prevalence also increased among both women and men in all education groups except men who were college graduates (Figure 2).

**FIGURE 1:**
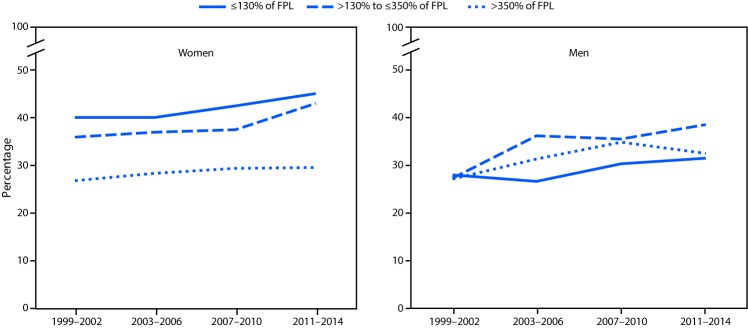
Obesity prevalence among adults, by household income (percentage of FPL) and sex — National Health and Nutrition Examination Survey, 1999–2002 to 2011–2014*^†^ **Abbreviation:** FPL = federal poverty level. * Estimates age-adjusted by the direct method to the 2000 projected U.S. Census population using the age groups 20–39, 40–59, and ≥60 years. ^†^ Significant linear trends for all groups except >350% of FPL for women. For >350% of FPL for men also significant quadratic trend. All p<0.05.

**FIGURE 2:**
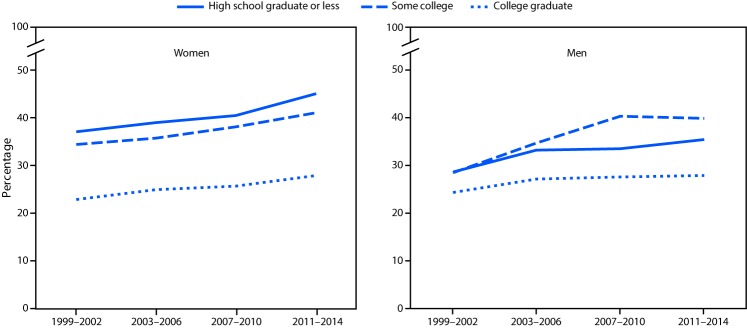
Obesity prevalence among adults, by education level and sex — National Health and Nutrition Examination Survey, 1999–2002 to 2011–2014*^†^ * Estimates age-adjusted by the direct method to the 2000 projected U.S. Census population using the age groups 20–39, 40–59, and ≥60 years. ^† ^Significant linear trends for all groups (p<0.01) except men who were college graduates. For women college graduates p = 0.056.

## Discussion

During 2011–2014, the relationships between obesity and income, and obesity and education were complex, differing among population subgroups. Whereas overall obesity prevalence decreased with increased levels of income and educational attainment among women, the association was more complex among men.

Similar to results based on data from 2005–2008 (*4*), during 2011–2014, obesity prevalence was lower in the highest income group among women, but this was not the case among men. In fact, among non-Hispanic black men the prevalence of obesity was higher in the highest income group than in the lowest income group. Both women and men who were college graduates, on the other hand, had lower prevalences of obesity than did persons with less education.

In general, prevalence of obesity among women was lowest among college graduates, although among non-Hispanic Asians there was no difference in prevalence by level of education. This relationship was not seen when obesity was examined by income level. For example, obesity prevalence was lower in the highest income group among non-Hispanic white women, but among non-Hispanic black women, prevalence did not differ between the highest and lowest household income groups. In contrast, among both non-Hispanic black women and non-Hispanic white women, the prevalence of obesity was lower among college graduates than among women with some college. This difference in the relationship between obesity and income and obesity and education has been reported in at least one other study (*7*) in children. These findings demonstrate that lower levels of income and education are not universally associated with obesity; the association is complex and differs by sex and race/Hispanic origin.

This is the first report to describe differences in obesity prevalence by income and education among non-Hispanic Asian adults. There were no significant differences in prevalence by income or education among either non-Hispanic Asian women or men; however, there was a pattern of decreasing prevalence with increasing income among non-Hispanic Asian women. 

The findings in this report are subject to at least two limitations. First, BMI is a proxy for body fat and BMI ≥30 was applied to persons in all racial/Hispanic origin groups, which might result in underestimating health risks for certain populations. For example, it has been suggested that the BMI cut point (≥30 kg/m^2^) that typically defines obesity might be too high for Asians and underestimate associated health risks (*8*,*9*). Second, the small sample size among some subgroups reduced the ability to detect differences when differences exist. Additional years of data might provide more information about obesity prevalence by income, especially among non-Hispanic Asian women.

Trends in obesity prevalence over time show that differences by income and education have existed at least since 1999–2002 among women. Among men, college graduates have consistently had a lower prevalence of obesity, whereas differences by household income have been less consistent. Further study is needed to understand the reasons for the different patterns by sex and race/Hispanic origin in the relationship between obesity and income or education.

**Summary**

**What is already known about this topic?**

Studies have suggested that obesity prevalence varies by income or education, although patterns might differ in high and low income countries.

**What is added by this report?**

Analysis of data from the 2011–2014 National Health and Nutrition Examination Survey (NHANES) examining the association between obesity and education and obesity and income among U.S. adults demonstrate that obesity prevalence patterns by income vary between women and men and by race/Hispanic origin. The prevalence of obesity decreased with increasing income in women (from 45.2% to 29.7%), but there was no difference in obesity prevalence between the lowest (31.5%) and highest (32.6%) income groups among men. Moreover, obesity prevalence was lower among college graduates than among persons with less education for non-Hispanic white women and men, non-Hispanic black women, and Hispanic women, but not for non-Hispanic Asian women and men or non-Hispanic black or Hispanic men. The association between obesity and income or educational level is complex and differs by sex, and race/non-Hispanic origin.

**What are the implications for public health practice?**

NHANES will continue to be an important source of data on disparities in obesity prevalence. These data will help track the *Healthy People 2020* objective of reducing obesity disparities and might inform CDC, state, or local obesity prevention programs.
